# MAD2L2, a key regulator in ovarian cancer and promoting tumor progression

**DOI:** 10.1038/s41598-023-50744-7

**Published:** 2024-01-02

**Authors:** Kejun Xu, Xiaojiao Zheng, Hongyan Shi, Jilan Ou, Huiqing Ding

**Affiliations:** Gynaecology and Obstetrics Department, The First Affiliated Hospital of Ningbo University, Ningbo, 315000 People’s Republic of China

**Keywords:** Cancer genomics, Gynaecological cancer, Tumour biomarkers, Cancer, Cell biology, Computational biology and bioinformatics

## Abstract

Ovarian cancer (OVCA), a prevalent gynecological malignancy, ranks as the fourth most common cancer among women. Mitotic Arrest Deficient 2 Like 2 (MAD2L2), a chromatin-binding protein and a component of DNA polymerase ζ, has been previously identified as an inhibitor of tumor growth in colorectal cancer. However, the roles of MAD2L2 in OVCA, including its expression, impact, and prognostic significance, remain unclear. We employed bioinformatics tools, Cox Regression analysis, and in vitro cell experiments to investigate its biological functions. Our findings reveal that MAD2L2 typically undergoes genomic alterations, such as amplifications and deep deletions. Moreover, we observed an overexpression of MAD2L2 mRNA in OVCA patients, correlating with reduced survival rates, particularly in those with Grade IV tumors. Furthermore, analysis of mRNA biofunctions indicated that MAD2L2 is predominantly localized in the organellar ribosome, engaging mainly in NADH dehydrogenase activity. This was deduced from the results of gene ontology enrichment analysis, which also identified its role as a structural constituent in mitochondrial translation elongation. These findings were corroborated by KEGG pathway analysis, further revealing MAD2L2’s involvement in tumor metabolism and the cell death process. Notably, MAD2L2 protein expression showed significant associations with various immune cells, including CD4+T cells, CD8+T cells, B cells, natural killer cells, and Myeloid dendritic cells. Additionally, elevated levels of MAD2L2 were found to enhance cell proliferation and migration in OVCA cells. The upregulation of MAD2L2 also appears to inhibit the ferroptosis process, coinciding with increased mTOR signaling activity in these cells. Our study identifies MAD2L2 as a novel regulator in ovarian tumor progression and offers new insights for treating OVCA.

## Introduction

Ovarian cancer (OVCA) is a common gynecological malignant tumor and the 4th most common tumor in women after breast cancer, cervical cancer, and endometrial cancer. In 2020, 313,959 and 55,342 patients were diagnosed with OVCA worldwide and in China, respectively. It is also the third highest female tumor-causing death (207,252 (4.7%) worldwide and 37,519 (18.10%) in China) after breast cancer and uterus cervical carcinoma^1,2^. The CONCORD 2018 trends researched the prevalence of OVCA in 61 countries and showed that the 5-year survival rate for OVCA from 2010 to 2014 was 30–50%, remaining the same as 1995–1999 worldwide. Furthermore, the 5-year survival rates for OVCA increased by 5% in the United States, Israel, South Korea, and parts of Europe, while in Japan, the number increased by 20% compared to 1995–1999 worldwide^3^. The OVCA incidence in China was not high, while its high mortality made OVCA to be regarded as one of the vital malignant tumors among Chinese females.

Individual precision therapy was currently the best option for patients first diagnosed with locally advanced and advanced ovarian cancer^4^. Alternatively, two novel exploratory studies were produced in 2022: The first study was a prospective, multicenter, Simon Phase 2 single-arm study of homologous recombination deficiency (HRD)-positive, advanced epithelial ovarian cancer conducted in China to evaluate the utilization of the poly ADP-ribose polymerase (PARP) inhibitor Nylappa in homologous recombination deficiency. The efficacy and safety data of the first 20 enrolled patients in this study were presented at the 2022 annual meeting of the Society of Gynecologic Oncology. A partial remission rate was achieved in 6 cases within 8 evaluable patients, and R0 resection was achieved in 4 of the 7 patients undergoing intermittent surgery in this study^5^. The first phase of the study has been completed, and further data are expected to be published soon. The second study is an open-label, multicenter pilot study to evaluate the efficacy and safety of olapalimab monotherapy (cohort 1) and olapalimab combined with pabolizumab (cohort 2) as a neoadjuvant in patients with HRD-positive ovarian cancer in stage III to IV. This study showed that the efficacy of Olapalib monotherapy in patients with HRD-positive advanced ovarian cancer was good, and the objective response rate, which was based on response evaluation criteria in solid tumor version 1.1, was 50%^6^. Preliminary studies of cohort 2 as a novel NACT are still ongoing. These studies suggested the critical role of PARP inhibitors in the clinical treatment of patients with unresectable OVCA as the latest advances.

PARP inhibitors showed a significant effect in OVCA treatment. However, a previous study indicated that the loss of MAD2L2 renders PARP inhibitors ineffective in BRCA1-deficient tumors, suggesting a new possible mechanism of MAD2L2 for the acquisition of resistance to PARP inhibitors in OVCA patients^7^, which makes the underlying mechanisms of MAD2L2 in OVCA worth to get a deep insight. Moreover, MAD2L2 was reported to be overexpressed in multiple cancers, such as glioma, breast cancer, and epithelial OVCA^8,9^. It has shown that MAD2L2 was involved in cell cycle regulation, DNA mismatch repair process, and cancer progression^10^. These findings hint at the critical role of MAD2L2 mechanisms in cancers. They might help in better cancer management in the future.

Meanwhile, immune inflammation level and the occurrence of tumors are closely related to prognosis in many types of cancer, which has been confirmed in many tumors^11^. Moreover, immune inflammation faction is an essential characteristic of cancer and plays a crucial role in the tumor microenvironment process. Studies have found that immune-related inflammatory factors are independent prognostic factors for overall survival (OS) and progression-free survival in epithelial ovarian cancer patients. Their predictive ability is significantly better than other inflammatory factors^12^. Consistently, BIZZARRI N et al. also confirmed that high levels of immune-inflammatory factors are associated with poorer OS in early-stage ovarian cancer patients. Additionally, it was also demonstrated that high levels of immune inflammation level are correlated with poorer 3-year disease-free survival (DFS) in early-stage ovarian cancer patients. The DFS was poorer in the subset of BRCA mutation patients with high levels of immune inflammation faction^13^.

Our study discovered that MAD2L2 is overexpressed in OVCA patients, correlating with decreased survival rates, particularly in those with Grade IV tumors. Remarkably, this research demonstrates for the first time that MAD2L2 is predominantly concentrated in the sulfur relay system and ribosome. Elevated levels of MAD2L2 enhance cell proliferation and migration in OVCA cell lines. Furthermore, the upregulation of MAD2L2 inhibits the ferroptosis process and is associated with mTOR signaling activity in these cells. Overall, our findings establish MAD2L2 as a significant regulator of tumor progression in ovarian cancer and contribute novel insights for OVCA treatment strategies.

## Results

### MAD2L2 was highly expressed in the tumor tissue of OVCA patients

**Figure 1:**
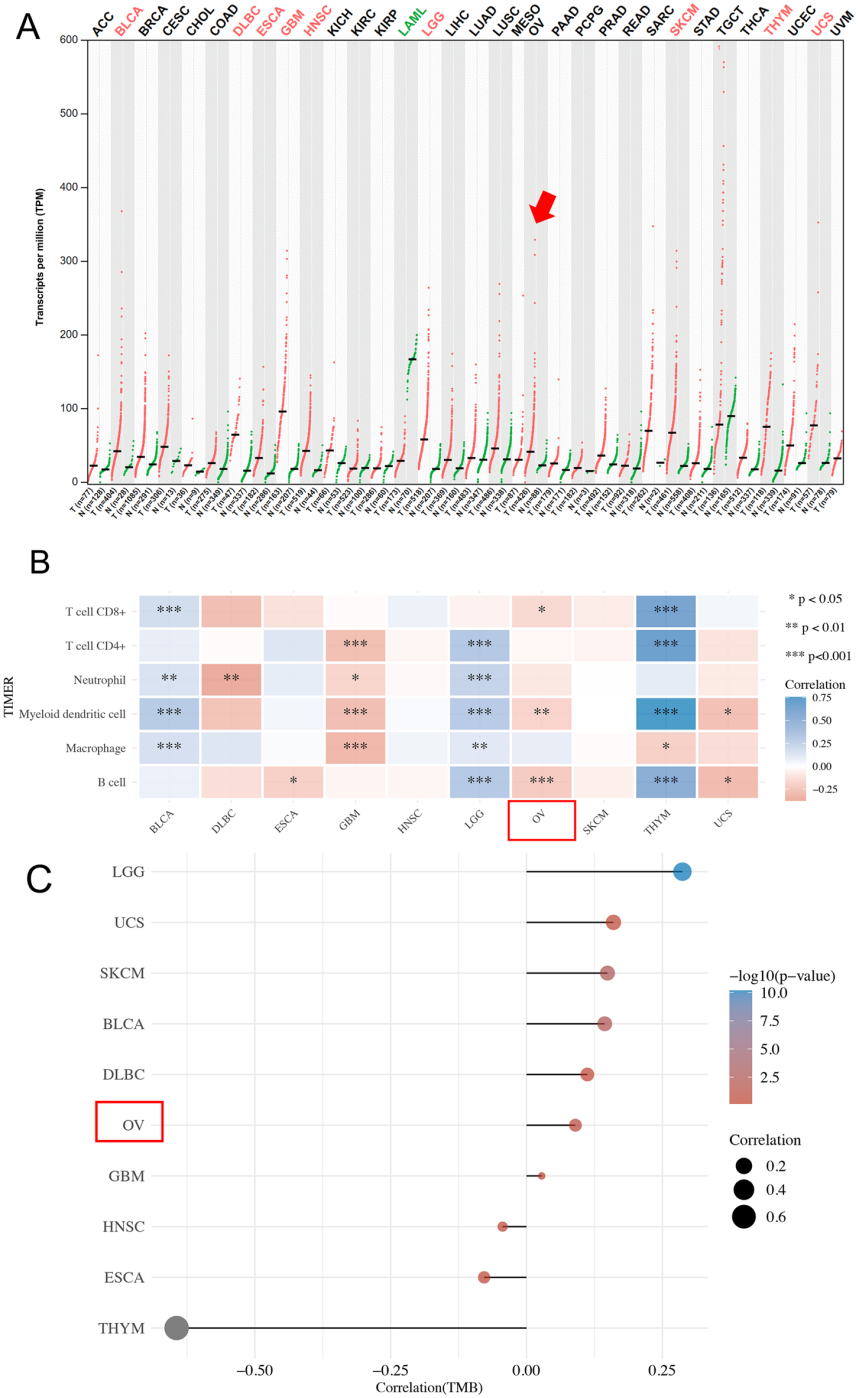
The gene expression of MAD2L2 in pan-cancer and survival time of five common cancers. (**A**) The expression of MAD2L2 across all tumor samples and paired normal tissue. T represents a tumor sample, and N represents a normal tissue sample. The red arrow indicates the OVCA group. The pan-cancer samples in red mean the tumor group, and in green mean the normal group. (**B**) The components of tumour infiltration immune cells within the 10 selected tumors and the relationship of the immune cells with the expression of MAD2L2. (C) The mutation of 10 tumors and the positive or negative relation of tumor cells with the expression of MAD2L2.

We initially applied the gene expression profiling interactive analysis 2 (GEPIA2) to determine the expression of MAD2L2 in pan-cancer sample data (Fig. 1A) between tumor samples and paired normal tissue samples taken from the same patient. We found that the MAD2L2 expression in tumor tissues was significantly higher than the paired normal tissues in pan-cancer samples (*P* value < 0.05). Secondly, we selected the top 10 highly MAD2L2-expressed tumors labeled in red in Fig. 1A, including OVCA and analyzed the levels of different tumor-infiltrating immune cells of the selected 10 tumors (Fig. 1B). The results showed that the expression of MAD2L2 in OVCA was negatively correlated with myeloid dendritic cells and B cells. However, it is highly expressed in various immune cells of thymoma (Fig. 1B). Finally, we calculated the tumor mutation burden (TMB) and found that the expression of MAD2L2 was highly associated with OVCA patients’ TMB, and the highest correlation ratio with MAD2L2 level was found in the low-grade glioma (Fig. 1C).

### High expression of MAD2L2 indicating poorer outcome in OVCA patients

**Figure 2:**
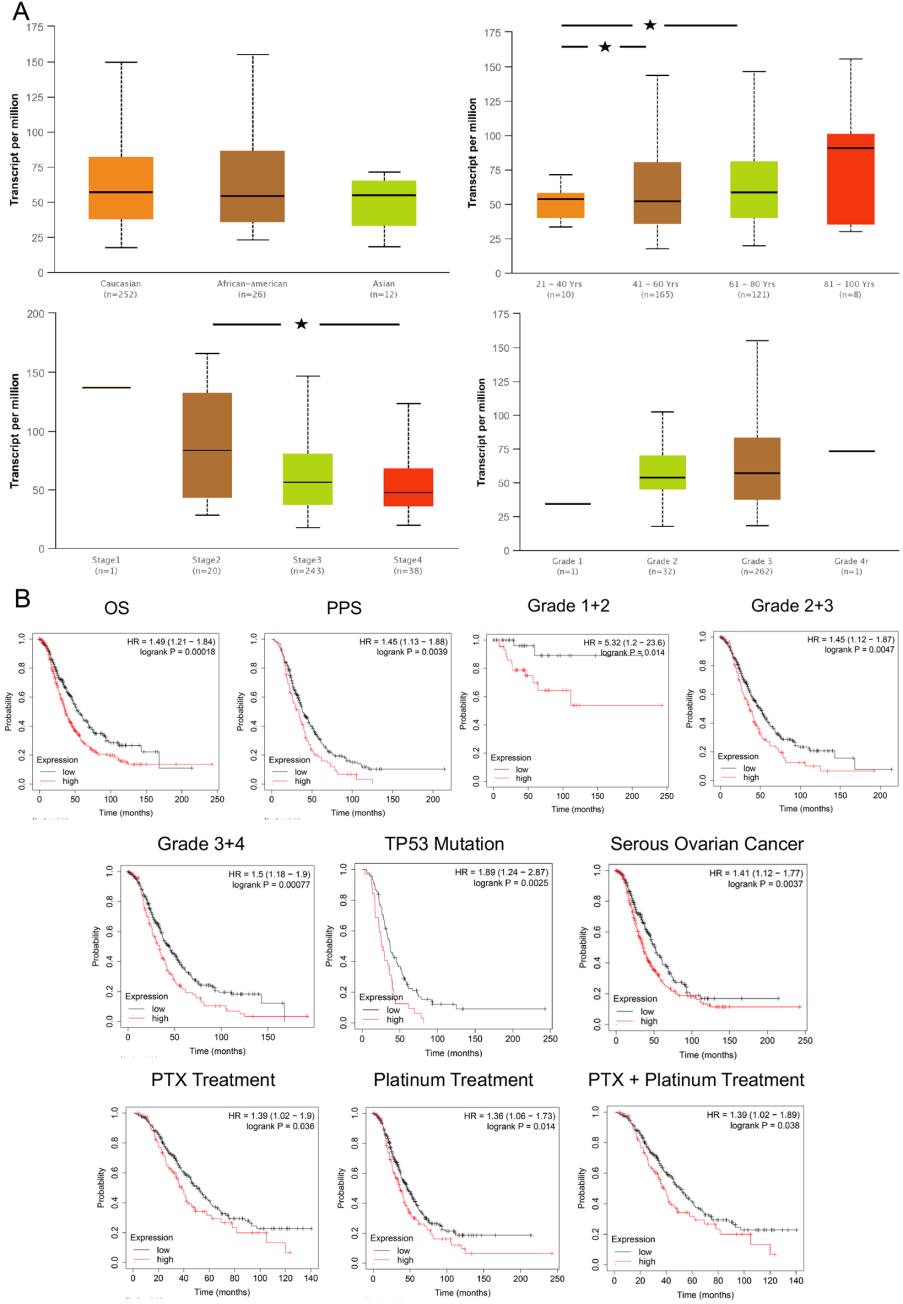
The clinical feature of MAD2L2 in OVCA patients. (**A**) The different clinical features of MAD2L2 in OVCA. (**B**) The survival time of MAD2L2 of OS, PPS, Grade1-4, TP53 mutation, Serous ovarian cancer, PTX, Platinum and PTX + Platinum in OVCA patients.

After finding that MAD2L2 was highly expressed in multi-tumor tissue, the clinical characteristics in OVCA patients were further evaluated. We found the expression of MAD2L2 was higher in the elderly patients and Caucasian patients (*P* value = 0.012 and 0.061) (Fig. 2A). Considering the clinical stage and grade, the expression of MAD2L2 was higher in Stage 2 and Grade 3 than other stage and grade groups (*P* value = 0.001 and 0.082) (Fig. 2A).

Furthermore, the overall survival (OS) and post-progression survival (PPS) data of MAD2L2-high OVCA patients were significantly shorter than MAD2L2-low patients (*P* value < 0.001 for OS, *P* value = 0.004 for PPS). Meanwhile, the OS of Grade 4 was significantly shorter than that of patients with the earlier grade in the MAD2L2-high group (*P* value = 0.00077 for Grade 4) (Fig. 2B). Besides, for TP53 mutation patients and serous ovarian cancer patients, the MAD2L2-high group were also having poor outcomes (*P* value = 0.00077 and 0.0037). In the drug treatment stage, three treatment methods, Paclitaxel (PTX), Platinum, and PTX + Platinum, all had poor outcomes for MAD2L2-high expressed OVCA patients.

### Frequency and type of MAD2L2 DNA alterations in OVCA patients

**Figure 3:**
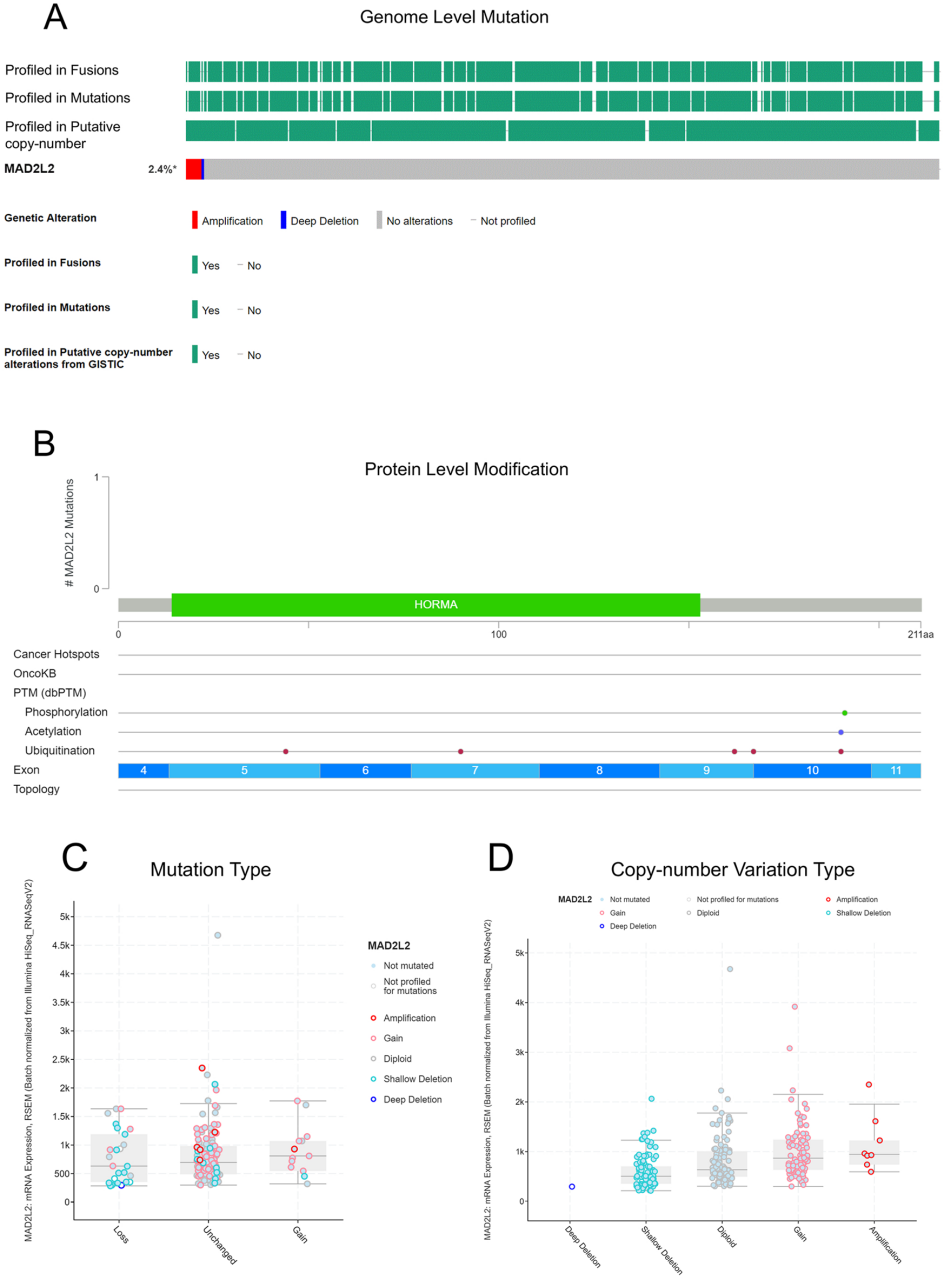
The gene alteration of MAD2L2 in OVCA. (**A**, **B**) The MAD2L2 genome level mutations and modification. (**C**, **D**) Boxplots showing the mutation types and copy-number variation types of MAD2L2.

Clinical data analysis has indicated that high expression of MAD2L2 could be found in large numbers of OVCA patients. Therefore, we employed the cBioPortal to detect the alternation types and frequency of MAD2L2 DNA in OVCA tumor samples based on the TCGA OVCA database. The frequency rate of the MAD2L2 alternations was 2.4% in OVCA patients (Fig. 3A), mainly amplifications and deep deletions. Amplifications were the main type of MAD2L2 copy number variation (CNV) in OVCA patients (*P* = 0.018). At the protein level, ubiquitination was the primary regulation way of MAD2L2 (Fig. 3B). At the same time, the gain was the highest mutation type and the CNV type of MAD2L2 in mRNA level (*P* value = 0.026) (Fig. 3C,D).

### Enrichment analysis of MAD2L2 mRNA functional networks in OVCA patients

**Figure 4:**
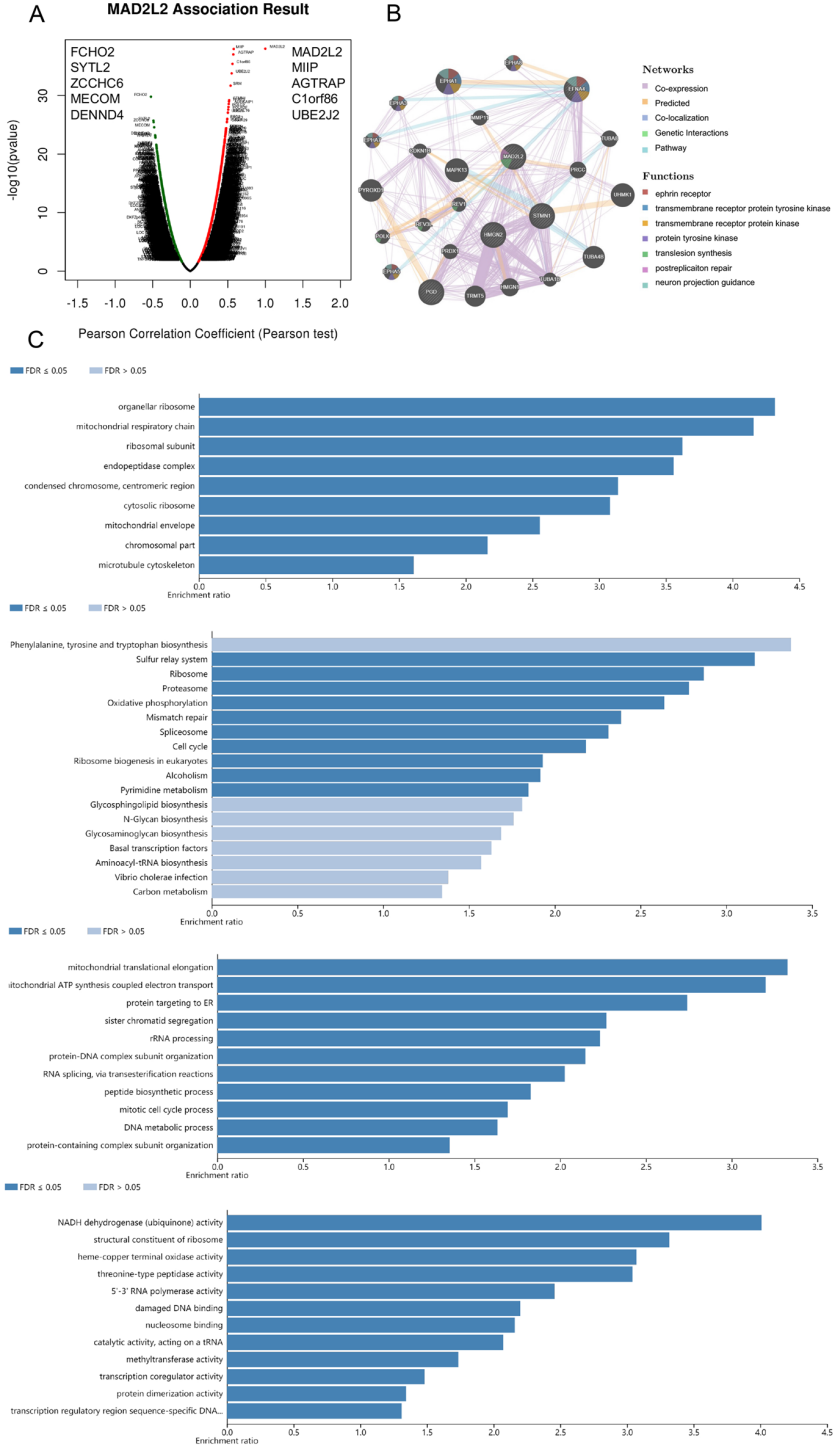
The gene enrichment analysis of MAD2L2. The enrichment analysis of MAD2L2. (**A**, **B**). Relative genes analysis, neighbor gene network, enrichment analysis and interaction analyses of MAD2L2 and protein–protein interaction network. (**C**). Bar plot of GO enrichment in cellular component, biological process, molecular function, and KEGG enriched terms.

Besides the above DNA data analysis, the cell bio-function module of LinkedOmics was used to analyze the mRNA level of MAD2L2 from OVCA patients in the TCGA. 784 genes (dark red dots) showed significant differential expressed when MAD2L2 was upregulated, whereas 538 genes (dark green dots) showed significant negative correlations (false discovery rate (FDR) < 0.01) (Fig. 4A). Additionally, correlative analysis indicated that the MAD2L2 expression showed a strong positive association with expression of PGD, STMN1, and EFNA4 (Fig. 4B). Significant GO term analysis from gene set enrichment analysis showed that MAD2L2 correlative and differentially expressed genes were located mainly in the organellar ribosome, mitochondrial respiratory chain, and ribosomal subunit during cell location analysis (Fig. 4C), In terms of biological processes, these genes were primarily involved in NADH dehydrogenase activity, structural constituent of ribosome and heme-copper terminal oxidase activity (Fig. 4C). Furthermore, They acted as regulators in mitochondrial translation elongation, ATP synthesis coupled with electron transport, and protein targeting to ER based on molecular function analysis (Fig. 4C). KEGG pathway analysis showed that MAD2L2 correlative genes were significantly enriched in the sulfur relay system, ribosome, proteasome and oxidative phosphorylation processes (Fig. 4C).

### Protein level expression and single cell level analysis in OVCA tissue

**Figure 5:**
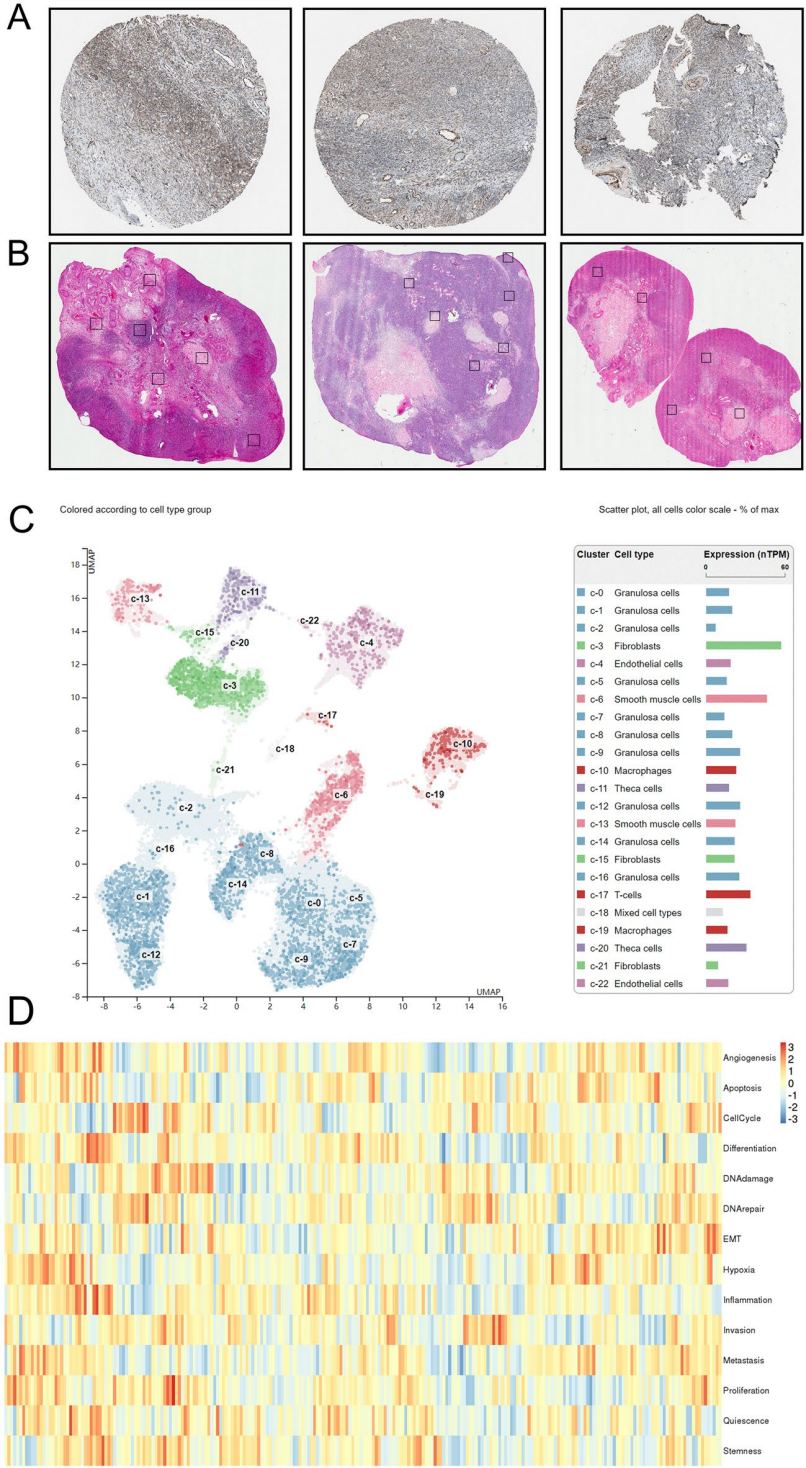
Protein level expression of MAD2L2 in single cell analysis and tumor samples. (**A**, **B**) IHC stained slides of MAD2L2 in OVCA, paracancerous tissue, and H&E staining, respectively. 40X indicates 40X magnification under an inverted microscope. (**C**, **D**) UMAP plot for the single cell RNA-seq analysis of OVCA patients’ samples and the correlation and bio-function between MAD2L2 high expression and different tumor environment cells.

At the protein level, immunohistochemistry (IHC), hematoxylin, and eosin (H&E) staining were employed to analyze MAD2L2 expression in OVCA patients. IHC slides displayed moderate expression of MAD2L2 in ovarian tumor samples from three female patients aged 33, 35, and 65 years (Fig. 5A). These slides revealed that both ovarian follicular cells and ovarian cancer stem cells exhibited moderate levels of MAD2L2 protein antibodies (stained blue), contrasting with the brownish-yellow staining of normal ovarian cells, highlighting their cytoplasmic and membrane localization. Additionally, H&E staining, as depicted in Fig. 5B, specifically highlighted ovarian cancer cells, indicated by black boxes.

Furthermore, single-cell protein level analysis was conducted using scRNA-seq data from OVCA tissue (GSE85534). This analysis revealed that granulosa cells were the predominant cell type among tumor-relative infiltrating cells in OVCA tissues. The highest expression of MAD2L2 was observed in fibroblasts, smooth muscle cells, and T cells, whereas the lowest expression was noted in granulosa and mixed cell types, compared to other cell types in the scRNA-seq samples (Fig. 5C). Additionally, scRNA-seq analysis revealed that cells with high MAD2L2 expression were significantly involved in angiogenesis, apoptosis, and the cell cycle (Fig. 5D).

### Tumor-infiltrating immune cells in OVCA patients

Apart from tumor cells, tumor micro-environment was also significant for tumor progress. The immune reaction in tumor tissue, such as the expression level of genes involved in inflammatory responses in OVCA cancer, might affect the clinical outcome of OVCA patients. To further explore the immune responses related to MAD2L2 in OVCA patients, we embarked on a comprehensive exploration of the correlation between MAD2L2 and immune cell infiltration using the EPIC, MCPCOUNTER, QUANTISEQ, and Immune Cell Infiltration (TIMER) database. Within EPIC, a positive correlation was shown between MAD2L2 expression and the infiltration of CD4 cells (*P* = 0.003) (Table 1). Within MCPCOUNTER, a positive correlation was shown between MAD2L2 expression and cytotoxicity score (*P* < 0.001) and NK cells (*P* = 0.002) (Table 1). Within QUANTISEQ, MAD2L2 expression was positively correlated with macrophage M1 cells (*P* = 0.007) (Table 1). Within TIMER, positive correlations were shown between MAD2L2 expression and B cells (*P* = 0.001), CD8 cells (*P* = 0.008), and dendritic cells (*P* = 0.004). (Table 1).

**Table 1:** The results of immune cell infiltration in patients with OVCA.

MAD2L2 expression			
	*P* value	R	n
EPIC			
B cell	0.837	− 0.01	263
CD4 + cell	**0.003**	− 0.18	263
CD8 + cell	0.082	0.11	263
Endothelial cell	0.647	− 0.03	263
Macrophage cell	0.395	− 0.05	263
NK cell	**0.030**	− 0.13	263
Uncharacterized cell	0.154	0.09	263
Mcp-counter			
T cell	0.953	0.00	263
CD8 + cell	0.207	0.08	263
Cytotoxicity cell	**4.46e**−**04**	− 0.22	263
NK cell	**0.002**	− 0.19	263
B cell	0.497	− 0.04	263
Monocyte cell	0.079	− 0.11	263
Macrophage cell	0.079	− 0.11	263
Myeloid dendritic cell	0.098	− 0.10	263
Neutrophil cell	0.837	0.01	263
Endothelial cell	0.948	0.00	263
Quantiseq			
B cell	0.111	− 0.10	263
Macrophage M1 cell	**0.007**	− 0.17	263
Macrophage M2 cell	0.113	− 0.10	263
Monocyte cell	**0.024**	− 0.14	263
Neutrophil cell	0.065	0.11	263
NK cell	0.509	0.04	263
CD4 + cell	0.179	− 0.08	263
CD8 + cell	0.452	− 0.05	263
T cell	0.108	− 0.10	263
Myeloid dendritic cell	0.087	0.11	263
Uncharacterized cell	**0.001**	0.20	263
TIMER			
B cell	**0.001**	− 0.20	263
CD4 + cell	0.631	0.03	263
CD8 + cell	**0.002**	− 0.19	263
Neutrophil cell	0.169	− 0.09	263
Macrophage cell	0.100	0.10	263
Myeloid dendritic cell	**0.004**	− 0.18	263

Bold numbers indicates the statistical difference by comparing with the other group.

### High level of MAD2L2 promoted cell proliferation and migration in OVCA patients

**Figure 6:**
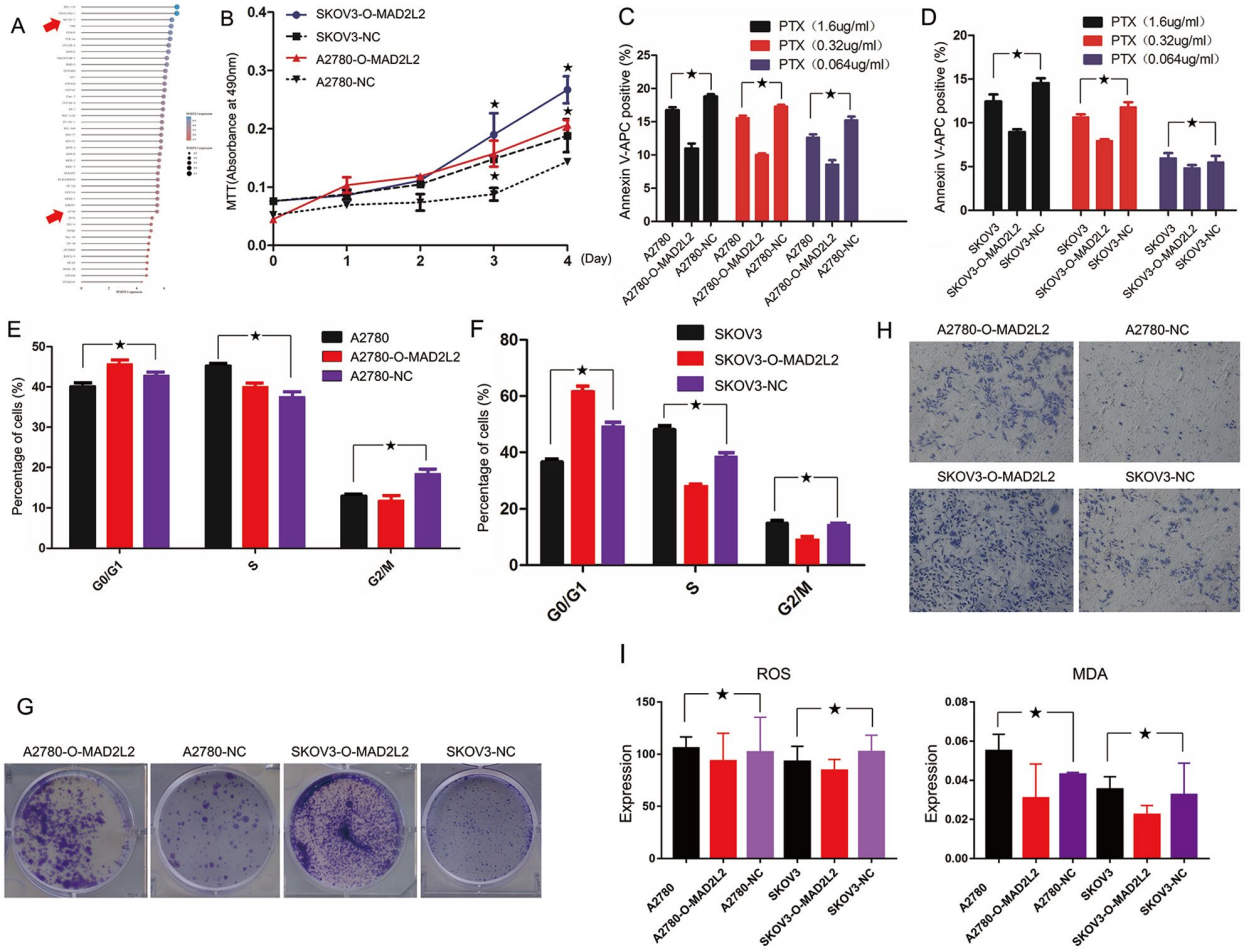
Overexpressed MAD2L2 promotes proliferation and the survival of OVCA cell lines, and overexpressed MAD2L2 promotes invasion in OVCA. (**A**) The expression of MAD2L2 in different OVCA cells. (**B**) Proliferation rates of NT, overexpression cells treated as indicated were measured by MTT assay (n = 5). The cell cycle distribution of each group cell was analyzed by flow cytometry. (**C**, **D**) Apoptotic level of each group in SKOV3 and A2780 was tested by flow cytometry (n = 3). (**E**, **F**) After 14 days, cells were stained with crystal violet and imaged to assess their colony formation ability. (**G**) After 14 days, cells were stained with crystal violet and imaged to assess their colony formation ability. (**H**) Matrigel invasion chambers were used to measure the invasiveness of each group cell treated as indicated, then stained with crystal violet and photographed. Magnification, ×200 for cells; scale bar, 100 μm. (**I**) The ROS and MDA level of MAD2L2 in OVCA cells.

To further verify the result achieved from the TCGA database, we overexpressed MAD2L2 by infecting human SKOVCA3 and A2780 cell lines with lentivirus-encoding RNA-targeting human MAD2L2. The upregulation of MAD2L2 increased the survival and proliferation capacities of SKOVCA3 and A2780 cell lines, as revealed by colony formation and MTT assays, respectively (Fig. 6A,B,G). Moreover, flow cytometry indicated that MAD2L2 rising stalled cell cycle progression in the G0/G1 phase in the SKOVCA3 cell line and the G0 phase in the A2780 cell line, too (Fig. 6E,F). Together, these findings suggested that the overexpressed MAD2L2 accelerated the proliferation and survival of OVCA cell lines in vitro by arresting cell cycle progression and inducing apoptosis. Besides, the trans-well invasion assays and the cell-extracellular matrix adhesion assay showed that the rising of MAD2L2 expression improves invasion in both SKOVCA3 and A2780 cell lines (Fig. 6H). Besides, the apoptotic level declined in cells of upregulating MAD2L2 under three concentrations of PTX in both cell lines (Fig. 6C,D). In addition, reactive oxygen species (ROS) assay and multiple discriminant analysis (MDA) showed that the upregulation of MAD2L2 was combinate with the lower level of oxidative stress process (Fig. 6I). All the results above indicated that the overexpressing of MAD2L2 accelerated OVCA cell motility in vitro and was consistent with the results got from the TCGA database.

### Upregulation of MAD2L2 Suppressed Ferroptosis Process through mTOR signaling in OVCA cell lines

**Figure 7:**
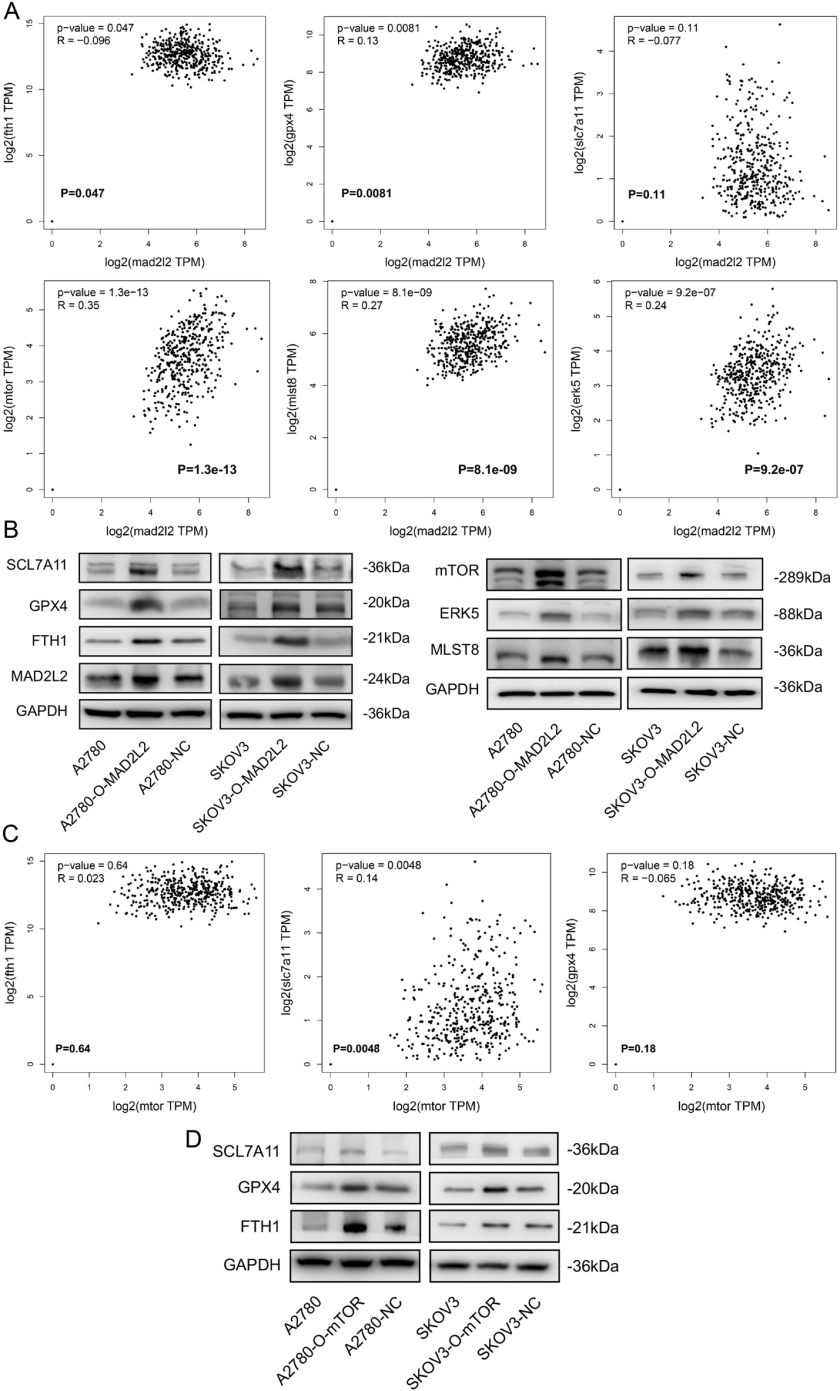
Upregulation of MAD2L2 Suppressed Ferroptosis Process and Accompanied by mTOR Signaling activity in OVCA cells. (**A**) The predictive correlation of MAD2L2 and ferroptosis relative genes/mTOR signaling relative proteins. (**B**) The Western blot results of MAD2L2 upregulation expression and ferroptosis relative proteins/mTOR signaling relative proteins. (**C**) The predictive correlation of mTOR and ferroptosis relative genes. (**D**) The Western blot results of ferroptosis relative proteins and overexpression of mTOR proteins.

At last, the above data analysis and cell experiments have validated the carcinogenesis of MAD2L2 in OVCA samples and cells. To explore the deep machine, the KEGG enrichment analysis and scRNA-seq relative bio-function evaluations were conducted and showed that MAD2L2 was significantly related to tumor cell metabolism, oxidative phosphorylation, and cell death. Therefore, we found that ferroptosis-relative genes’ mRNA expressions were significantly higher in MAD2L2-high OVCA tissues (Fig. 7A), especially SLC7A11, GPX4, and FTH1 were highly expressed in the MAD2L2 over-expressed SKOVCA3 and A2780 cell lines by Western blot experiments (Fig. 7B). In this part, we concluded that MAD2L2 might downregulate ferroptosis process in the progression of OVCA cells, while the regulation process being direct or indirect deserves future examine.

To further analyze how MAD2L2 suppresses ferroptosis, we found in the literature that the protein level of mTOR, ERK5, and MLST8 was upregulating in over-expressed MAD2L2 OVCA cells (Fig. 7B). Furthermore, the prediction results from TCGA database presented that mRNA expression of mTOR was critically correlated with SLC7A11 (Fig. 7C). Besides, the Western blot results were consistent with the prediction data, which meant that in the MAD2L2 over-expressed OVCA cells, mTOR might upregulate SLC7A11 to restrain ferroptosis and promote tumor growth in OVCA cells (Fig. 7D).

## Discussion

Our study indicates that MAD2L2 may play a carcinogenic role in OVCA, potentially serving as a predictor for clinical prognosis. MAD2L2, also known as hRev7 or MAD2B, is a highly conserved protein. Prior research has highlighted its critical function in the anaphase cell process, ensuring proper chromosome alignment at the metaphase plate and involvement in other DNA repair processes^14^. Additionally, numerous studies have reported dysregulation of MAD2L2 and its related proteins, such as AURKB, in various cancers^15^. MAD2L2 is implicated in response to DNA damage, potentially acting downstream of RIF1, contributing to genomic instability, a critical factor in cancer initiation^16^.

In OVCA, DNA damage events are a primary cause of carcinogenesis and treatment failure. Recent research suggests that reduced MAD2L2 expression may increase cisplatin sensitivity in ovarian clear-cell carcinoma cells^17^. Additionally, Sergey Karakashev et al. discovered that inhibition of EZH2 upregulates MAD2L2, potentially enhancing the efficacy of PARP inhibitors in CARM1-dependent OVCA^18^. Furthermore, Abdolrahim Abbasi et al. proposed that mutations in MAD2L2 might contribute to developing OVCA in a mouse model^19^.

Previous studies have revealed that the expression of mTOR proteins is abnormal in OVCA. A report at the 2016 ASCO meeting showed a cohort of 379 patients with ovarian or fallopian tube carcinoma have been evaluated for 10 genes AKT1, AKT2, AKT3, mTOR, PIK3CA, PIK3C2B, PIK3R1, PTEN, TSC1, TSC2 in the PI3K/AKT/mTOR pathway^20^. Among the 379 patients, 33% had at least one gene mutation. The mutation rate of mTOR was 12%, indicating that the occurrence of OVCA was closely related to the activation of the PI3K/AKT/mTOR pathway^21^. Statistics show that the PI3K/AKT/mTOR signaling pathway is activated in about 70% of OVCA patients. Under the action of PI3K, AKT, and mTOR inhibitors, the proliferation of cancer cells was blocked, and apoptosis was increased. For instance, Pi-103 is a double inhibitor of PI3K and mTOR, and PI-103 can significantly increase the sensitivity of SKOV3/DDP (OVCA drug-resistant cell line) cell lines to cisplatin and induce cell cycle arrest and apoptosis by inhibiting PI3K/Akt/mTOR signaling pathway^22^. Therefore, blocking the PI3K/AKT/mTOR signaling pathway may become a new target for the OVCA treatment. In this research, we found that mTOR, ERK5, and MLST8 protein levels were upregulated in overexpressed MAD2L2 OVCA cell lines.

Among them, mTOR is a serine/threonine protein kinase that plays a vital role in cell growth and can sense signals such as energy, nutrition, growth factors, and hormones inside and outside the cell. mTOR is composed of two complexes: mTOR complex 1 (mTORC1) and mTOR complex 2 (mTORC2)^23^. mTORC1 differs from mTORC2 in that mTORC1 contains rapamycin-sensitive protein, which makes it sensitive to rapamycin, while mTORC2 does not. Besides, their different compositions also cause different functions: mTORC1 can promote mRNA translation, protein synthesis and degradation, lipid synthesis, energy metabolism, and participate in autophagy. mTORC1 synthesizes protein through the S6K1 and 4E-BP112 pathways and degrades protein through the NRF1 pathway to maintain the protein in the cell in a balanced state. Meanwhile, mTORC2 can activate AKT by phosphorylating Ser473 of AKT, acting on downstream mTORC1 and promoting the growth of tumor cells^24^. At last, we first reported the relationship between the role of MAD2L2 and mTOR signaling in OVCA, which can provide new ideas and insights for the clinical treatment of OVCA and Nylappa resistance in the future.

## Conclusions

Our study has established that dysfunction in the DNA mismatch repair process in OVCA cells is closely linked to tumor progression, particularly influencing tumor growth and invasion. Within this context, MAD2L2, a mismatch repair protein, emerges as a critical gene and a potentially valuable clinical biomarker for survival prognosis. Our research also indicates a correlation between the increased tumorigenic capacity of OVCA cells and elevated mTOR signaling, coupled with reduced ferroptosis activity. Consequently, targeting MAD2L2 could represent an alternative therapeutic strategy for OVCA, especially beneficial for patients who do not respond effectively to PARP inhibitors.

## Materials and methods

### Data source

MAD2L2 expression and clinical data of TCGA pan-cancer data and GTEx were obtained from the GEPIA2 database (http://gepia2.cancer-pku.cn/#dataset). To evaluate MAD2L2 expression, tumor tissues were obtained from TCGA, and normal tissues were combined from the TCGA and GTEx databases. Ovarian cancer data were collected from the GEO database, including GSE12470 (Platform: GPL887) and GSE40595 (Platform: GPL570), and used for RNA-Seq Version 2 (RNAseqV2) exon sequencing data and miRNA sequencing (miRNA-Seq) data analysis.

### Bioinformatics analysis

This study performed MAD2L2 mRNA expression analysis of tumor and normal tissues, pathological stage analysis, and correlative prognostic analysis of MAD2L2 with the analysis module of GEPIA2 (http://gepia2.cancer-pku.cn/#dataset) database^25^. Clinical relative expression data of MAD2L2 was obtained using the “Expression Analysis” module of UALCAN (https://ualcan.path.uab.edu)^26^. Genetic alterations, co-expression, and network modules of different genes could be obtained through the cBioPortal (www.cbioportal.org/) module, among which mRNA expression of z scores (RNA Seq V2 RSEM) was worked out using a z score threshold of ± 2.0. Meanwhile, protein expressions of z scores (RPPA) were also obtained using a z score threshold of ± 2.0^27^. GeneMANIA (http://genemania.org/) was utilized to analyze the connection between our research genes of OVCA cancer and others^28^. DAVID 6.8 (https://david.ncifcrf.gov)^29^ was employed in this research, and the functions of the Gene Ontology (GO) enrichment analysis, biological processes, cellular components, molecular function, and Kyoto Encyclopedia of Genes and Genomes (KEGG) pathway enrichment analysis of submitted genes and closely related neighbor genes were applied using the “Express Analysis” module of Metascape (www.metascape.org/)^30^ was applied to verify the enrichment of submitted genes and closely related neighbor genes. “Gene module” of TIMER (http://timer.cistrome.org/)^31^ was used to evaluate the correlation between MAD2L2 gene level and the infiltration degree of immune cells. The cell expression analysis of MAD2L2 was done by Human Protein Atlas (www.proteinatlas.org/) to compare it with normal tissue and check the survival curve of tumor patients^32^. The single-cell sequencing data was analyzed using UMAP of R package Seurat for nonlinear cluster analysis. Then, through the clustering results, gene expression levels were calculated to identify each cell type and label each cell type with a different color.

### Survival analysis

Cox regression can assess the correlation between the expression of 30k genes (mRNA, miRNA, protein) and survival in 25k+ samples from 21 tumor types, including breast, ovarian, lung, gastric cancer, etc. This study performed the prognostic analysis of MAD2L2 using Kaplan–Meier curves^33^.

### Cell culture

Our lab cultured human ovarian cell lines SKOV3 and A2780 in RPMI 1640 and DMEM (Invitrogen) with 10% FBS (Gibco, Grand Island, USA). All cell lines were obtained from the cell bank of the Chinese Academy of Sciences (Shanghai, China) and authenticated by DNA fingerprinting. Also, all cell lines were maintained under standard cell culture conditions at 37°C and 5% CO2 and passaged for < 2 months after resurrection.

### Reagents and antibodies

The following primary antibodies were used in this study: SLC7A11 (DF12509), GPX4 (DF6701), FTH1 (DF6278), MAD2L2 (DF12288), mTOR (AF6308), ERK5 (DF6835), MLST8 (df6204), PRAS40 (DF8396) and GAPDH (AF7021). The following reagents were used: lentiviral vectors CMV-MCS-3FLAG-SV40 and pLOV-CMV-eGFP (Gene, Shanghai, China), lipofectamine 2000 (Invitrogen).

### Western blot

After collection, cells were lysed in a lysis buffer on the ice and supplemented with phenylmethylsulfonyl fluoride, a phosphatase inhibitor, and a protease inhibitor cocktail. Western blot was carried out as described in the instruction. To analyze the relative protein level of interest, the band grayscale of interested protein and loading control was quantified using the analysis software, and the ratio was calculated and correlation tested.

### Colony formation assay

Cells were seeded in triplicate at 200 cells/well in 6 well plates and cultured for 10 days. Then, they were washed three times with PBS, fixed in methanol for 30 min, dyed with crystal violet for 30 min at room temperature, and washed out the dye with pure water later. The plates were photographed, and the colonies' numbers were compared and statistically analyzed using the test.

### MTT assay

Cells were seeded on a 96-well plate at 1 × 104 cells/well in RPMI 1640 containing 10% FBS. Each sample had three replicates. The medium was replaced every other day. Viable cells were counted by the MTT assay. Briefly, cells were incubated with 50 μl of 0.2% MTT for half an hour at 37°C in the 5% CO2 incubator. The absorbance at 560 nm was determined using the 96-well plate reader (Dynex Technologies), and the results were analyzed using a correlation test.

### Flow cytometry assay

Cells were cultivated for 24 h. The harvested cells were washed with PBS and fixed with ice-cold 70% ethanol overnight. The fixed cells were resuspended in PBS (containing 0.2 mg/mL RNAse and 10 mM PI) and incubated in the dark for 30 min at 37 degrees. A FACSCalibur flow cytometer (Becton Dickinson, San Jose, CA) was used for flow cytometric analysis, and the results were analyzed using a correlation test.

### Trans-well and invasion assay

Matrigel invasion chambers (8 μm, 24-well cell culture inserts) were used according to the manufacturer’s protocol for the cell invasion assay. 5 × 104 cells were resuspended in 500 μl RPMI1640 or DMEM and added to the upper chamber; 500 μl DMEM with 10% FBS was used in the lower chamber as a chemoattractant. After 22 h, cells on the upper surface of the membrane were removed using a cotton swab, and the membranes were stained with crystal violet. Cell numbers were determined by averaging cell counts from nine separate 10× fields. For the trans-well assay, the Matrigel was removed, and the experimental procedure was followed as described above.

*ROS and MDA array* The experiment was performed per the kit instructions.

### Statistical analyses

Visualization was done using the “ggplot2” package. Immune analysis was performed, and the “ggstatsplot” package was utilized to accumulate the correlations between gene expression and immune score. The “pheatmap” package was used to draw multi-gene correlation. Besides, Spearman’s correlation analysis described the correlation between quantitative variables without a normal distribution^34^. We used the statistical language R (version 4.1.1) to calculate the statistics. Intracranial tumor volumes are expressed as mean ± standard error of the mean. All other values are expressed as mean ± SD of a minimum of three independent biological replicates. The dissimilarities between the two groups were established using Student's t-test, while the dissimilarities among multiple groups were established using one-way analysis of variance (ANOVA). Overall survival curves were analyzed using the Kaplan–Meier method and compared through a log-rank test. Spearman's rank correlation was used to evaluate the associations between two variables. The figures indicate statistical significance at *P* < 0.05 (*), *P* < 0.01 (**), *P* < 0.001 (***), and *P* < 0.0001 (****).

### Ethics approval and consent to participate

OVCA cells were collected from the Chinese Academy of Sciences (Shanghai, China). The study was conducted per the Declaration of Helsinki. Bioinformatic analysis was obtained from the Ethics Committee of the Ningbo First Hospital.

### Supplementary Information

Supplementary Information.

## Data Availability

The datasets used and/or analyzed during the current study are available from the corresponding author upon reasonable request.
