# Supplementary figures and images for: MAD2L2, a key regulator in ovarian cancer and promoting tumor progression
Source: Sci Rep. 2024 Jan 2;14:130. doi: 10.1038/s41598-023-50744-7 (PMC10761867; doi:10.1038/s41598-023-50744-7)

**Cell Information**

1. SKOV3 cell

STR information:


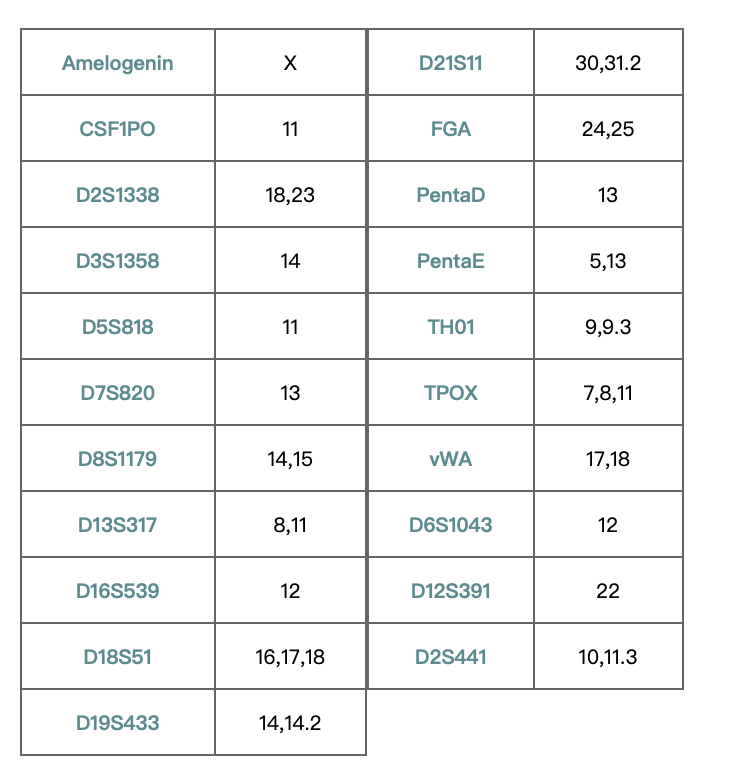


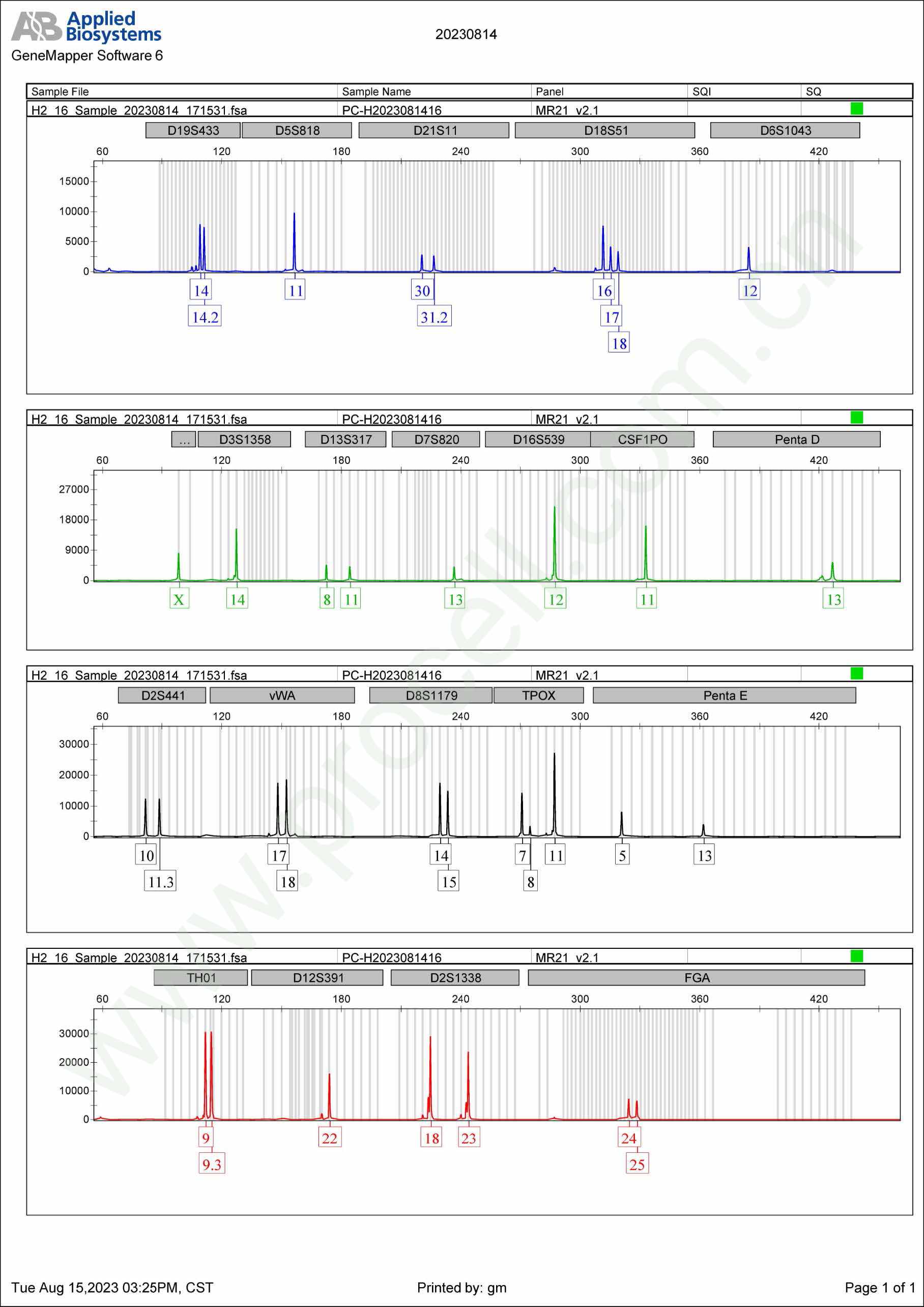


1. A2780 cell


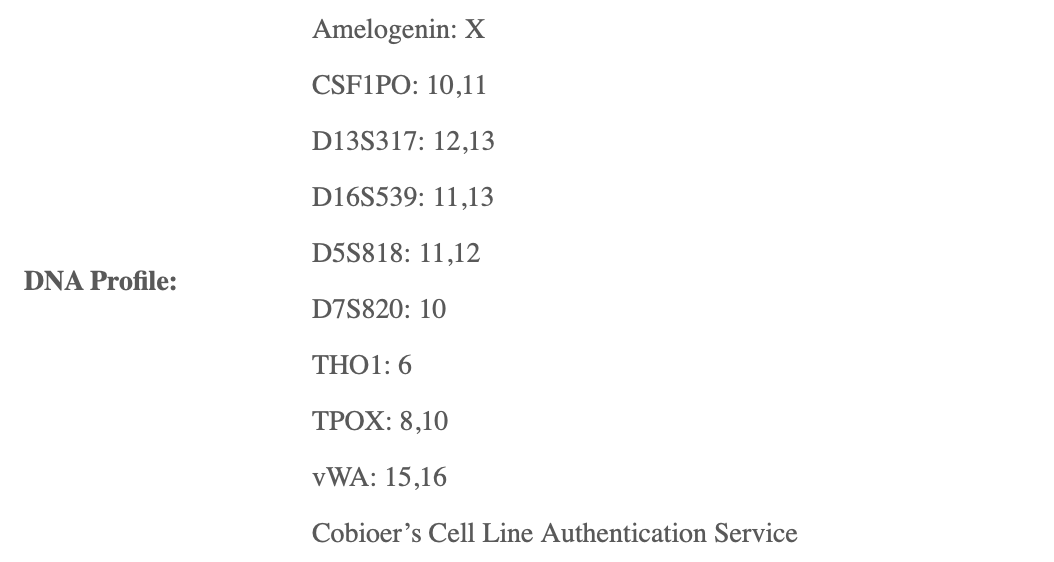


STR information:


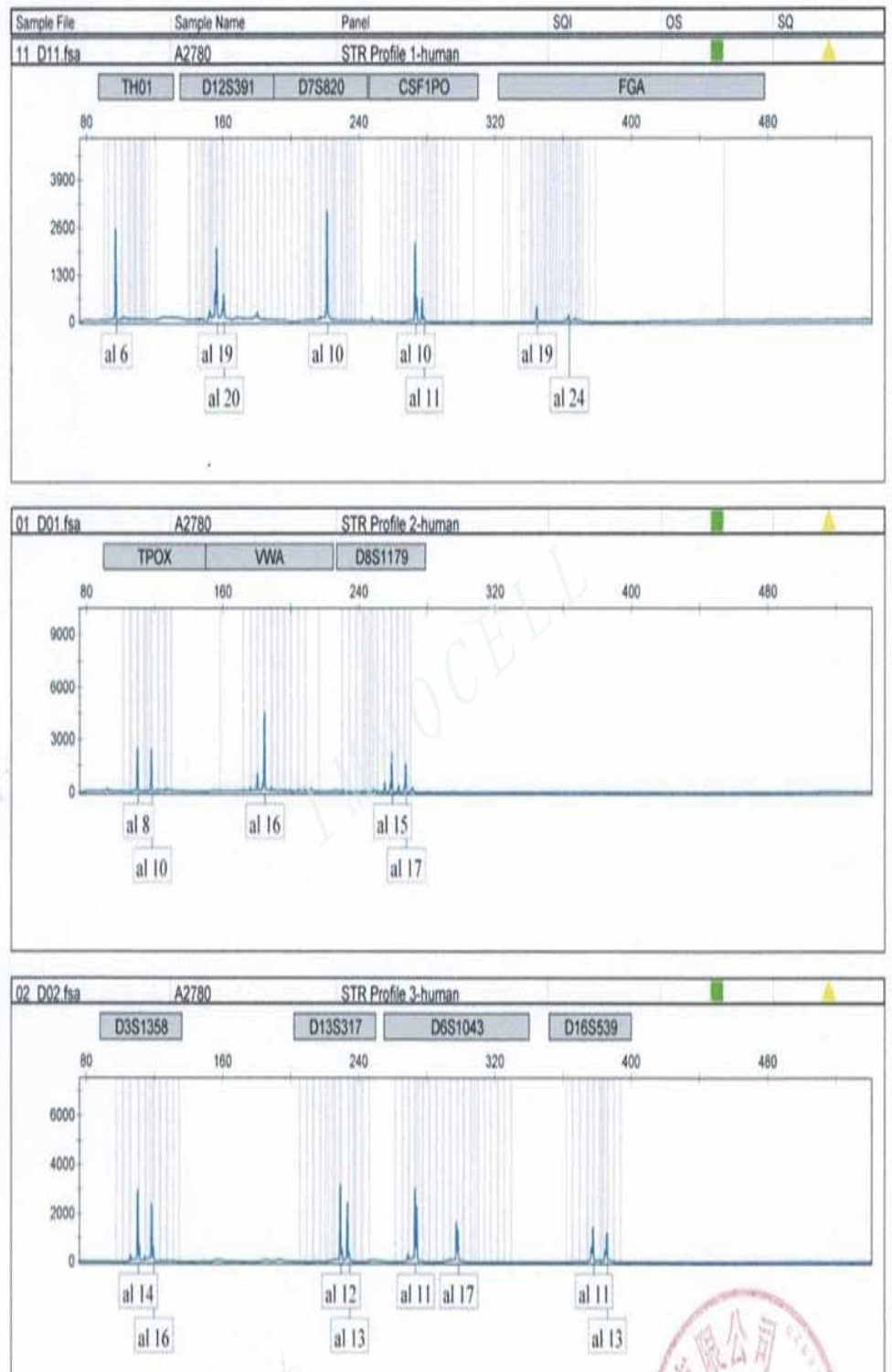

Supplement: Supplementary file 1 — Supplementary Information. [file 41598_2023_50744_MOESM1_ESM.docx]
